# Is the goal of 12,000 steps per day sufficient for improving body composition and metabolic syndrome? The necessity of combining exercise intensity: a randomized controlled trial

**DOI:** 10.1186/s12889-019-7554-y

**Published:** 2019-09-03

**Authors:** Tsung-Lin Chiang, Chu Chen, Chih-Hsiang Hsu, Yu-Chin Lin, Huey-June Wu

**Affiliations:** 1 Graduate Institute of Sport Coaching Science, Chinese Culture University, Taipei, Taiwan; 2 Department of Physical Education, National Taiwan Normal University, Taipei, Taiwan; 3 Physical Education Office, National Taipei University of Business, Taipei, Taiwan; 4 Department of Combat Sports and Chinese Martial Art, Chinese Culture University, Taipei, Taiwan

**Keywords:** Walking, Daily step goal, Step rate, Moderate intensity, Cardiovascular disease risk factor

## Abstract

**Background:**

To investigate the differences in body composition and metabolic syndrome (MS) under a daily 12,000-step strategy with or without moderate-intensity walking exercise in college students with obesity.

**Methods:**

Thirty-two adults with obesity (mean (s.d.) age: 19.72 (0.80) years; height: 165.38 (3.99) cm; wt: 83.31 (4.66) kg; body mass index: 30.38 (0.83) kg m^− 2^) were recruited and randomly assigned to the walking step goal group (WSG; achieving 12,000 steps per day), walking exercise group (WEG; achieving 12,000 steps per day, including 3 days per week on which walking at a step rate of over 103 steps min^− 1^ was required), or control group (CG; maintaining a free-living life style). Each participant’s accumulated daily steps from daily activities and walking exercises were monitored using a smartwatch for 8 weeks. The variables of body composition and MS were measured before and after intervention.

**Results:**

Average daily steps over 8 weeks did not significantly differ between the WSG and WEG (11,677.67 (480.24) vs. 12,131.90 (527.14) steps per day, respectively, *P >* .05). Although the CG and WSG showed no improvement in body composition, the WEG exhibited significant improvements in terms of hip circumference and visceral fat area (VFA) (∆ − 2.28 (3.27) cm and ∆ − 13.11 (9.83) cm^2^, respectively, *P* < .05); high-density lipoprotein cholesterol (HDL-C), fasting glucose (FG), and triglycerides (TG) (∆ 16.36 (8.39), ∆ − 2.53 (3.73), and ∆ − 10.52 (36.26) mg dL^− 1^, respectively, *P* < .05). The WSG exhibited improvements only in HDL-C (∆ 14.24 (16.13) mg dL^− 1^, *P* < .05).

**Conclusion:**

The combination of walking exercise program and daily step goal is a more time efficient strategy in improving body composition and MS than simply establishing a daily step goal. Furthermore, this strategy may also include a potential reduction effect on the risk factors of cardiovascular diseases.

**Trial registration:**

Australian New Zealand Clinical Trials Registry, number ACTRN12618001237279 (Retrospectively registered).

## Background

Obesity is a global concern because of its causative role in various diseases. Overweight and obesity increase the risk of developing cardiovascular diseases (CVDs). Additionally, some risk factors of CVDs are collectively referred to as metabolic syndrome (MS); MS is defined by the presence of any three of the following risk factors: abdominal obesity, hypertension, elevated blood glucose, elevated triglycerides (TGs), and reduced high-density lipoprotein cholesterol (HDL-C) [1]. A study on Taiwanese individuals from 2005 to 2008 determined the prevalence of overweight and obesity during this period was 50.8 and 36.9% among men and women, respectively, and MS was 25.5 and 31.5% among men and women, respectively [2]. A study on the period 2013–2014 revealed the prevalence of obesity in men and women was 48.9 and 38.3%, respectively [3]. These studies have revealed overweight and obesity are common among the Taiwanese population; reducing their prevalence depends on effective monitoring and treatment to reduce their impact on human health.

A sedentary lifestyle is a key factor of MS morbidity. Ford, Kohl, Mokdad, and Ajani recruited 1626 adults (aged ≥20 years) to investigate associations among sedentary behavior, physical activity, and MS. The results indicated the risk of developing MS increased 1.41- and 2.10-fold when the adults’ sedentary lifestyles increased by more than 1 and 4 h a day, respectively (odds ratio [OR] = 1.41 and 2.10, respectively) [4]. Therefore, reducing the number of sedentary hours and increasing physical activity frequency are effective for MS prevention.

Walking is a straightforward method for increasing physical activity and is not limited by location. Studies have demonstrated people who walk between 10,000 and 12,000 steps per day generally have a lower body mass index (BMI), body fat percentage, waist and hip circumference, and waist–hip ratio [5–7]. Sisson et al. revealed MS prevalence decreased as one’s daily steps increased; specifically, the odds of having MS were 10% lower for each additional 1000 steps per day (OR = 0.90) [8].

In previous studies, whether daily step goals were gradually implemented over the interventions period (incremental approach to achieving 10,000 steps per day over 12 weeks) or fully implemented all at once (10,000 steps per day), the MS and body composition outcomes of the interventions remain equivocal [9, 10]. Some studies have been unable to demonstrate expected improvement effects possibly because using a step goal as the only criterion was insufficient when other variables such as activity frequency, duration, and intensity were uncontrolled. Pal, Cheng, and Ho gave women with obesity a daily 10,000-step goal or invited them to engage in a 30-min walking exercise intervention for 12 weeks [11]. The results indicated body composition and blood pressure remained unchanged. Although exercise frequency and duration were considered, results diverged from the authors’ expectations [11]. Studies on regular moderate-intensity walking exercise have reported both continuous and intermittent walking exercises have positive effects on body composition and MS [12–14]. Based on the cited studies, we speculated that regular moderate-intensity walking exercise interventions are more effective than step goal strategies and that structural exercise programs are essential to exercise effectiveness. To date, few studies have examined the effects of combining these two strategies. Therefore, this study investigated the effect of combining step goal strategies with supplemental exercise programs in accordance with exercise recommendations from the American College of Sports Medicine (ACSM) to more effectively understand the effects of these two strategies on body composition and MS in college students with obesity.

## Methods

In this study, 32 participants with obesity aged 18 years or older who did not regularly engage in physical activity were recruited. The inclusion criterion for participants were no diabetes or other chronic diseases. Approval was obtained from the Institutional Review Board of Fu Jen Catholic University (No. C105137). Prior to pre-intervention testing and following an information session, all participants provided written informed consent.

### Study design

This study included a pre-intervention measurement, 8-week intervention, and post-intervention measurement. 32 participants were randomly assigned to the walking step goal group (WSG), walking exercise group (WEG), or control group (CG). The grouping methods was confidential to the participants. All measurements and exercise intervention were completed in the campus of Chinese Culture University.

### Body composition and MS biomarker pre-measurement and post-measurement

The pre-measurement and post-measurement protocols were identical and completed within 7 days; they included weight (wt), BMI, body fat (FAT), visceral fat area (VFA), and skeletal muscle mass (SMM) and were measured using a body composition analyzer (Inbody 720, Biospace Co., Ltd., Seoul, ROK). Waist circumference (WC) and hip circumference (HIP) values were averaged based on the results of two time measurements with a tolerance error of 1 cm. Prior to measuring resting heart rate (HR), systolic blood pressure (SBP), diastolic blood pressure (DBP), and blood biomarkers, participants were asked to refrain from intense activity, smoking, caffeine consumption, and avoid the foods rich in sugar and fat for 24 h and to fast for 12 h before blood sampling. All resting values were obtained after a 10 min seated resting. A sample of approximately 5 ml of venous blood was drawn from each participant between 8 a.m. and 10 a.m. The samples were centrifuged at 3000 rpm for 10 min and subsequently stored in a − 80 °C environment. HDL-C, fasting glucose (FG), and TG levels were determined from the blood samples.

### Eight-week step goal and walking exercise intervention

All groups (WSG, WEG, and CG) were required to attend a procedure instruction session before the intervention and were issued a smartwatch (ZenWatch 3, ASUSTeK Computer Inc., Taipei, Taiwan) for step monitoring during the 8-week intervention. Three groups conducted the instruction session on different days. Participants from WEG and WSG must report back to the lab weekly for verification of any missing or incorrect data recording on the exercise log.

The specific intervention procedures for the 8-week intervention are described as follows: the WSG was asked to reach 12,000 steps per day from Monday to Friday. The WEG combined the aforementioned step goal with walking exercises; the participants were asked to walk 12,000 steps from Monday to Friday, including 30 min of continuous moderate-intensity (i.e., 103 steps min^− 1^) walking exercises on 3 days per week [15]. During the exercises, participants were monitored by professional instructors in order to maintain a steady brisk walking pace. The Borg’s rating of perceived exertion (RPE; scale 6–20) was used to assess the subjective perception of effort at the pre- and immediately post-exercise. The CG was not given any instructions regarding exercise during the intervention and was asked to maintain a similar daily routine including diet. However, the specific option of food was not controlled for all groups. We believed this will be closer to the actual daily-living scenario. For daily routine monitoring, smartwatch was used as active monitoring and exercise log as passive. Participants required to put on the smartwatch at the non-dominant wrist right after awakening and recorded the time of awakening on the exercise log. All participants have full access to the accumulated total steps on the smartwatch. Smartwatch was removed at the bedtime. The total steps should be recorded on the exercise log by the time just right before sleep.

### Data and statistical analysis

Data were analyzed using SPSS Statistics 22.0 (IBM Corporation, Armonk, NY, USA). Step rate [(pre-exercise steps – post-exercise steps)/total exercise duration] was used to define exercise intensity during the WEG’s 30-min moderate-intensity walking exercise. Each participant’s percentage changes in body composition and MS markers were calculated using the following formula: [(post-measurement – pre-measurement)/pre-measurement] × 100%. All descriptive and statistical data are shown as mean (s.d.). A paired-sample *t* test was used to compare the differences between pre-measurement and post-measurement body composition, MS markers, and RPE. Additionally, Cohen’s effect sizes (ES) were conducted to evaluate the magnitude of the change in body composition and MS markers following experimental protocols with the criteria of ≤0.49, small; 0.50–0.79 medium, and ≥ 0.80, large [16]. Independent samples analyses of variance (one-way ANOVAs) were conducted to compare the effects of the intervention on the mean steps per day over 8 weeks, body composition, MS markers, and percentage changes among groups. Post hoc analyses were conducted using Scheffé’s method. The partial eta squared (η $\frac{2}{p}$) was used to assess the effects size from ANOVA analyses. As per Cohen’s suggestion, 0.01 is considered a “small” effect size, 0.03 represents a “medium” effect size, and 0.14 is a “large” effect size [17]. The significance level was set at *P* < .05.

## Results

### Subjects

In this study, 32 subjects were recruited; 9, 12, and 11 were randomly assigned to the CG, WSG, and WEG, respectively (age: 19.36 (1.12) vs. 19.17 (1.03) vs. 20.64 (1.80) years, respectively). The pre-measurement results are presented in Tables 1 and 2.

**Table 1:** Pre-measurement and post-measurement data and percentage changes in body composition and resting HR

Variable	Group	Pre	Post	∆ Chang (%)	ES (95% CI)
Weight (kg)	CG	88.40 (8.93)	88.73 (8.80)	+ 0.41 (1.51)	0.34 (− 6.18 to 5.41)
	WS	82.28 (14.75)	82.52 (14.47)	+ 0.43 (3.47)	0.12 (−8.47 to 8.06)
	WE	79.25 (8.20)	78.37 (9.30)	−1.19 (3.50)	0.48 (−4.36 to 5.98)
BMI (kg/m2)	CG	31.02 (3.34)	31.13 (3.23)	+ 0.38 (2.08)	0.22 (−2.41 to 1.89)
	WS	30.68 (3.53)	30.65 (3.37)	+ 0.02 (3.22)	0.03 (−1.96 to 1.94)
	WE	29.45 (1.86)	29.07 (2.25)	−1.29 (3.83)	0.49 (−0.60 to 1.82)
HIP (cm)	CG	109.08 (8.82)	109.56 (7.53)	+ 0.59 (4.53)	0.14 (−5.91 to 4.78)
	WS	109.96 (7.64)	109.13 (7.97)	−0.77 (1.66)	0.68 (−3.64 to 5.19)
	WE	108.52 (5.55)	106.02 (6.05)	−2.28 (3.27)	0.97 (− 2.30 to 4.55)*
WHR	CG	0.90 (0.05)	0.89 (0.06)	−1.30 (4.60)	0.42 (0.38 to 0.45)
	WS	0.86 (0.05)	0.86 (0.08)	−0.49 (5.70)	0.14 (0.11 to 0.18)
	WE	0.92 (0.16)	0.84 (0.07)	−6.95 (10.00)	0.98 (0.88 to 1.02)
FAT (%)	CG	33.68 (7.96)	34.03 (7.22)	+ 1.75 (7.57)	0.22 (−5.42 to 4.50)
	WS	37.53 (7.89)	38.26 (7.48)	+ 2.30 (5.33)	0.54 (−5.00 to 3.70)
	WE	38.38 (6.06)	37.86 (6.09)	−1.38 (4.60)	0.45 (−3.13 to 4.05)
SMM (kg)	CG	32.90 (3.95)	32.94 (4.03)	+ 0.16 (3.62)	0.05 (−2.63 to 2.58)
	WS	28.61 (7.25)	28.48 (7.34)	−0.46 (2.83)	0.24 (−3.86 to 4.39)
	WE	27.15 (4.79)	27.05 (4.77)	−0.28 (3.65)	0.13 (−2.70 to 2.95)
VFA (cm^2^)	CG	96.83 (29.11)	95.83 (31.24)	−0.97 (12.81)	0.10 (−18.91 to 20.51)
	WS	101.60 (36.89)	100.53 (34.04)	−0.18 (8.89)	0.17 (− 20.70 to 19.43)
	WE	93.05 (18.98)	80.32 (15.54)	−13.11 (9.83)‡	2.08 (−9.14 to 11.26) *
Resting heart rate (bpm)	CG	79.78 (7.85)	73.67 (7.81)	−7.08 (11.61)	0.91 (−4.23 to 6.01)
	WS	75.92 (10.13)	75.58 (7.73)	+ 0.51 (11.52)	0.05 (−5.68 to 4.42)
	WE	78.73 (10.02)	70.91 (7.82)	−9.05 (12.11)	1.04 (−4.89 to 5.65)*

* P < .05: significantly different from pre-measurement

^‡^ *P* < .05: significantly different from CG and WSG. 95% confidence interval of the mean absolute difference between the groups

**Table 2:** Pre-measurement and post-measurement data and percentage changes in MS

Variable	Group	Pre	Post	∆ Chang (%)	ES (95% CI)
WC (cm)	CG	98.00 (7.28)	97.18 (9.17)	−0.81 (6.58)	0.18 (−4.57 to 6.18)
	WS	94.58 (9.44)	93.63 (13.47)	−1.31 (6.20)	0.33 (− 5.01 to 7.95)
	WE	99.82 (17.81)	89.25 (7.30)	− 9.11 (10.70)	1.10 (− 9.43 to 5.41)
SBP (mmHg)	CG	127.00 (17.18)	122.33 (13.50)	−2.14 (17.04)	0.31 (−10.91 to 9.13)
	WS	121.92 (15.70)	119.42 (10.90)	−1.53 (5.55)	0.74 (−8.15 to 6.90)
	WE	121.36 (11.48)	115.09 (10.45)	−4.63 (10.30)	0.69 (−6.09 to 6.87)
DBP (mmHg)	CG	74.33 (11.06)	71.78 (9.11)	−2.12 (15.34)	0.30 (− 6.92 to 6.25)
	WS	76.92 (12.06)	77.17 (9.21)	+ 1.16 (9.84)	0.05 (−6.87 to 5.17)
	WE	79.55 (8.85)	73.45 (8.85)	−6.67 (14.47)	0.73 (−4.50 to 5.95)
FG (mg dl^−1^)	CG	95.22 (7.31)	92.78 (6.38)	−2.21 (8.31)	0.43 (−4.34 to 4.60)
	WS	95.33 (5.42)	94.25 (5.71)	−1.07 (4.27)	0.38 (−2.69 to 3.61)
	WE	92.00 (6.60)	89.55 (5.26)	−2.53 (3.73)	1.05 (−2.86 to 4.15)*
TG (mg dl^−1^)	CG	103.89 (30.45)	91.89 (22.77)	−10.25 (13.35)	1.26 (−18.64 to 16.14)*
	WS	113.17 (58.73)	96.00 (39.13)	−6.98 (33.47)	0.75 (−32.48 to 22.89)
	WE	85.09 (39.63)	69.36 (27.05)	−10.52 (36.26)	1.13 (−22.29 to 17.12)*
HDL-C (mg dl^−1^)	CG	46.44 (9.91)	46.78 (7.81)	+ 1.78 (10.30)	0.10 (−6.58 to 5.00)
	WS	43.33 (7.18)	49.33 (10.15)	+ 14.24 (16.13)	1.24 (−5.30 to 4.51)*
	WE	45.55 (8.15)	53.09 (10.66)	+ 16.36 (8.39)‡	3.35 (−8.17 to 2.95)*

* P < .05: significantly different from pre-measurement

^‡^ *P* < .05: significantly different from the CG. 95% confidence interval of the mean absolute difference between the group

### Steps per day for each group and moderate-intensity steps rate for the WEG over 8 weeks

**Fig. 1:**
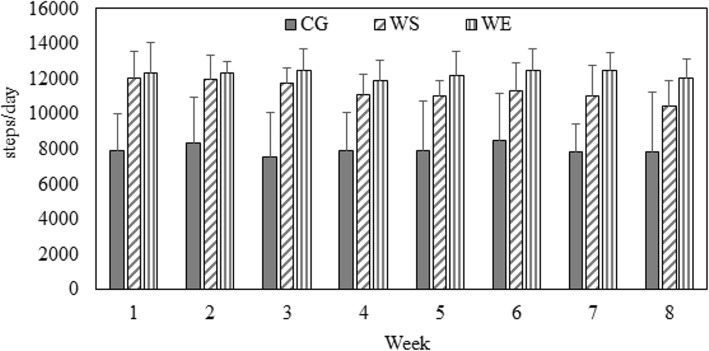
Average Total Daily Steps in Each Group over 8 Weeks

**Fig. 2:**
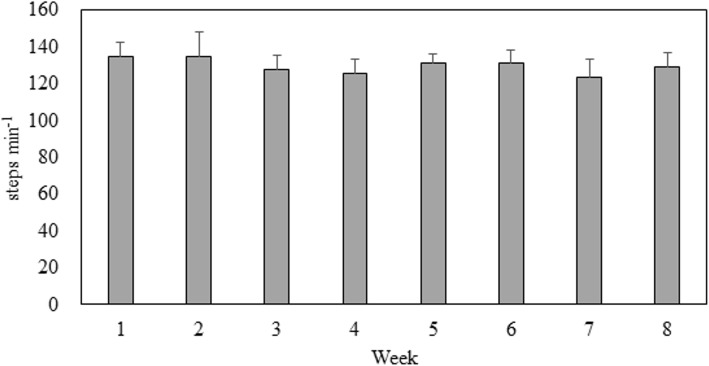
Walking Exercise Step Rate in the WEG over 8 Weeks

**Fig. 3:**
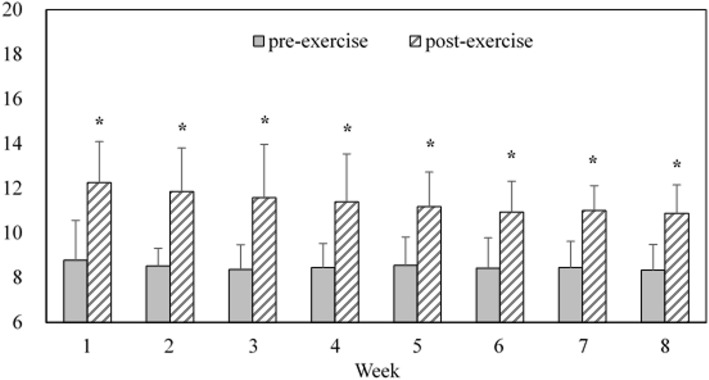
RPEs of Pre- and Post-Walking Exercise in WEG over 8 Weeks *P < .05 post-exercise significantly greater than pre-exercise

Figure 1 presents the average daily steps for each group over the 8-week intervention period (CG: 7977.74 (2174) vs. WSG: 11340.46 (743) vs. WE: 12288 (721) steps per day). Significant differences were observed among the groups (*F* = 29.334, η $\frac{2}{p}$ = 0.67, *P* < .05). Post hoc analysis revealed no differences in daily steps between the WSG and WEG (*P* > .05); however, daily step count in these two groups was significantly higher than in the CG (95% confidence interval (CI) 1882.21 to 4843.23; 2801.93 to 5820.08, *P* < .05). Figure 2 presents the step rate for the WEG’s walking exercise over 8 weeks. During the 30 min walking exercise of each week, the mean RPEs of post-exercise were significantly higher than pre-exercise over 8 weeks. (ES = 1.85 ~ 2.53, *P* < .05). Details are presented in Fig. 3.

### Differences between pre-measurement and post-measurement and percentage changes

Statistical analyses revealed pre-measurement body composition variables were not significantly different among the three groups (*P* > .05). Additionally, the pre-measurement and post-measurement body composition variables for the CG and WSG were not significantly different (*P* > .05). However, post-measurement hip circumference and VFA in the WEG were significantly lower than the corresponding pre-measurement values (ES = 0.97, 2.08 *P* < .05). Additionally, only the WEG exhibited a significant post-intervention reduction in resting HR (ES = 1.04, *P* < .05). Regarding percentage changes among all variables, only VFA differed significantly among the groups (*F* = 5.288, η $\frac{2}{p}$ = 0.27, *P* < .05); post hoc analysis revealed VFA in the WEG was significantly lower than in the WSG and CG (95% CI − 24.15 to − 1.71; − 24.23 to − 0.62%, *P* < .05). Details are presented in Table 1.

Regarding the pre-measurement values of all MS level variables, no significant differences were observed among the three groups (*P* > .05). Regarding pre-measurement and post-measurement differences, the post-measurement TG level was significantly lower than the pre-measurement TG level in the CG (ES = 1.26, *P* < .05). The post-measurement HDL-C level was significantly higher than the pre-measurement HDL-C level in the WSG (ES = 1.24, *P* < .05). In the WEG, the FG and TG levels were significantly reduced after the intervention (ES = 1.05; 1.13, *P* < .05), whereas the HDL-C level had significantly increased (ES = 3.35, *P* < .05). Additionally, the ANOVAs revealed changes in HDL-C level differed significantly among the three groups (*F* = 3.944; η $\frac{2}{p}$ = 2.14, *P* < .05). Post hoc analysis revealed the HDL-C level in the WEG was significantly higher than in the CG (95% CI 0.28 to 28.90%, *P* < .05). Details are presented in Table 2.

## Discussion

The effects of walking exercise on body composition and MS depend on the characteristics of intervention. A previous study established that walking 10,000–11,700 steps per day should be sufficient for patients with obesity to meet the physical activity guideline [1, 18]. Furthermore, studies have reported the negative consequences of unhealthy body composition and MS are reversed when patients walk 10,000–12,000 steps per day [5–7, 19, 20]. Therefore, the present study established 12,000 steps as a daily step goal to ensure that during the intervention, each participant walked a number of steps in accordance with those recommended by previous studies. The results indicated the WSG and WEG walked an average of 11,340 and 12,288 steps per day, respectively, during the 8-week intervention. The steps accumulated by both groups corresponded to step ranges recommended by previous studies and did not significantly differ from each other. Under these circumstances, the intervention had a more favorable effect on the WEG than the WSG, possibly because exercise intensity was greater for the WEG participants. Studies have reported the critical role of exercise intensity for affecting body composition and MS. To facilitate health improvements, engaging in regular moderate-intensity physical activity is essential [1, 21–24]. Hence, step rate seemed a more useful variable than daily step count for regulating the WEG’s walking exercise intensity during the intervention. This finding corresponds to those of previous studies have documented the effectiveness of step rate for estimating exercise intensity (*R*^2^ = .70–.91) [25, 26]. Furthermore, previous studies have reported the step rate range for moderate-intensity (three metabolic equivalents) walking exercise is 103–110 steps min^− 1^ [25, 26]. The average step rate of the WEG over the 8-week intervention was 129 steps min^− 1^, which exceeded the aforementioned requirement for moderate-intensity exercise. By contrast, the daily step goal for the WSG allowed for self-selected pace. A study on the daily walking exercise of 3744 adults reported a step rate range of 1–59 steps min^− 1^ was adopted by participants for an average of 8.7 h per day, whereas a step rate of > 100 steps min^− 1^ was adopted for an average of only 7 min per day, which is a low proportion for the > 100 steps min^− 1^ rate when pace is self-selected [27]. Thus, we speculated the WSG’s step rate was unable to meet moderate-intensity walking exercise requirements.

The WEG’s exercise intensity was likely higher than the WSG during the intervention. After regular moderate-intensity walking exercise, the WEG exhibited a lower resting HR (9.05%) and lower SBP (6 mmHg) and DBP (6 mmHg), whereas the WSG exhibited only a 2-mmHg reduction in SBP. Previous studies have revealed HR and blood pressure are direct markers for assessing exercise benefits. Regular exercise at an appropriate intensity may lead to a resting HR reduction and reductions in SBP and DBP by approximately 5–7 mmHg [28–30]. Tjønna et al. [23] found SBP and DBP decreased by 10 and 6 mmHg, respectively, after a 70–90% HR_max_ regular exercise intervention. The cited studies have clarified that engaging in regular exercise of adequate intensity generally accompanies cardiovascular response reductions. Therefore, in the present study, the WEG exhibited a greater reduction in cardiovascular responses than did the WSG, likely because of more intense stimulation.

The average daily steps in the CG and WSG were 7977 and 11,340, respectively. The WSG’s physical activity level was significantly higher than the CG; however, body composition remained unchanged after intervention. A similar result was reported after a 6–12-week intervention where average daily steps were between 8796 and 12,635; however, no improvement in body composition was observed. Nevertheless, significant reductions in body wt, BMI, body fat percentage, body fat mass, and hip circumference were observed when the intervention period was increased to 20–36 weeks. Furthermore, the improvement was even more pronounced when adherence was maintained for 36 weeks [10, 11, 20, 31]. The lack of improvement in the WSG in terms of body composition may be attributable to a slow self-selected pace and low-intensity walking exercise. In cases of insufficient exercise intensity, a prolonged intervention period may be necessary to ensure improvement. The present study attempted to more thoroughly understand whether walking exercise performed at a specified exercise intensity is the optimal method for obtaining health benefits. Although the WEG and WSG walked an approximately equal number of steps per day, the WEG exhibited more improvements in terms of hip circumference and VFA. Murphy et al. determined overweight adults exhibited significantly reduced hip circumference after performing one long bout (30 min) or three short bouts (10 min) of moderate-to-vigorous intensity (70–80% HR_max_) walking per day over a 6-week period [12]. Additionally, a previous study confirmed that a hip muscle group (e.g., gluteus medius) was critical for generating support and forward progression when walking, especially during the single-limb standing phase [32]. Hence, the hip circumference reduction effect in the WEG in this study may be attributable to the long-term training effect of walking in the aforementioned hip muscle group. An improvement in VFA was confirmed by Ayabe et al., who identified a negative relationship between bouts of physical activity of moderate-to-vigorous intensity and VFA. The results indicated MVPA lasting longer than 1 min and 3 min were significantly associated with VFA (r = −.328; −.382, respectively) [33]. We speculated the improvement in VFA in the WEG was attributable to continuous 30-min moderate-intensity walking exercise. However, the WEG did not exhibit any improvements in body wt, BMI, waist-to-hip ratio (WHR), body fat, and SMM. The ACSM’s Guidelines for Exercise Testing and Prescription state that adults with obesity or overweight must perform exercise of moderate-to-vigorous intensity for 50–60 min per session to maintain a long-term wt-loss effect [1]. A related intervention study found after a moderate-intensity walking exercise intervention that mandated three exercise sessions per week for 12 weeks, no improvements in body composition were observed regardless of whether participants walked continuously for 20 min or walked 2 × 10 min sessions [34]. However, body composition variables significantly improved after a moderate-intensity walking exercise intervention involving walking three times a week for 60–120 min each time for 12 weeks [13]. Despite regular moderate-exercise-intensity stimulation, the duration of the single-bout exercise of the WEG was lower than recommended. This may explain the limited improvement in body composition.

Regarding the MS benefits, although the WSG exhibited a significant increase only in HDL-C level, the WEG exhibited more evident improvements in MS variables, including FG, TG, and HDL-C. One study reported participants who walked 10,000 steps per day for up to 36 weeks exhibited significant improvements in WC and HDL-C, thereby further revealing the positive relationship between daily steps and HDL-C (*r* = .451; *P* = .007) [20]. Our results were also similar to an earlier study: participants regularly performed moderate-to-vigorous exercise three to five times per week, and significant improvements in FG, TG, and HDL-C were observed in Week 6 [12, 13]. However, in the present study, the CG exhibited a significant reduction in TG similar to the WEG; this may be attributable to dietary variations during the intervention. A previous study reported TG level was significantly affected by diet [35]. However, the present study did not record daily food intake during the intervention. TG reduction was difficult to identify from the effect of exercise intervention or diet. Hence, the causal relationship between exercise and TG reduction could not be established. Dietary monitoring should be included in future studies to clarify the effect of exercise on TG. Studies have revealed exercise intensity is critical for improving aerobic fitness and mitigating MS risk factors [23]. The reduced cardiovascular profile in the WEG may be a consequence of aerobic fitness improvement after regular moderate-intensity walking exercise. Related studies have reported a significant relationship between VFA and MS [36, 37]. A literature review confirmed VFA is related to TG, FG, and HDL-C [38]. Therefore, the VFA reduction (− 13.11%) in the WEG might accompany reductions in FG, TG, and HDL-C.

This study combined an identical step goal strategy with moderate-intensity walking exercise. Cardiovascular responses (resting HR and blood pressure) were significantly reduced by increasing the step rate in the walking exercise intervention based on suggestions on moderate-intensity exercise (> 103 steps min^− 1^). Furthermore, VFA, blood lipid profile, and hip circumference all exhibited significant improvement after intervention. The current results suggest that combining a step goal with a moderate-intensity walking exercise is an effective strategy for ensuring exercise benefits and shortening the duration required to improve body composition and MS.

## Conclusion

Current results show that moderate-intensity exercise should be performed in combination with a daily step goal to ensure health benefits from walking exercise in adults with obesity. Additionally, step rate is an exercise intensity variable that can be precisely managed. Although step goal strategy increased the physical activity effectively, the effect on health benefits was limited. It might be the shortcoming of simply using step goal as a health improvement strategy. The present study demonstrates the necessity of incorporating exercise prescription guideline (FITT) with the walking exercise program, which using step goal as a cardiovascular and metabolic syndrome improving strategy.

However, the current results are limited to adults with obesity. Additional investigations concerning different populations, or a greater age range are required. To more thoroughly understand why the most body composition and some MS variables did not improve following the intervention, we suggest a prolonged intervention period (> 8 weeks), more frequent exercise (5 days per week), or a longer single-session duration (50–60 min per session) be investigated in future research.

## Data Availability

The datasets analysed during the current study are available from the corresponding author on reasonable request.

## Abbreviations

ACSM: American College of Sports Medicine
BMI: Body mass index
CG: control group
CI: Confidence interval
CVDs: Cardiovascular diseases
DBP: Diastolic blood pressure
ES: Effect size
FAT: Body fat
FG: Fasting glucose
HDL-C: High-density lipoprotein cholesterol
HIP: Hip circumference
HR: Resting heart rate
MS: Metabolic syndrome
RPE: Rating of perceived exertion
SBP: Systolic blood pressure
TGs: Triglycerides
VFA: Visceral fat area
WC: Waist circumference
WEG: walking exercise group
WSG: Walking step goal group
